# Dietary supplementation of essential oils in dairy cows: evidence for stimulatory effects on nutrient absorption

**DOI:** 10.1017/S1751731118001696

**Published:** 2018-07-20

**Authors:** H.-S. Braun, K. T. Schrapers, K. Mahlkow-Nerge, F. Stumpff, J. Rosendahl

**Affiliations:** 1 Institute of Veterinary Physiology, Faculty of Veterinary Medicine, Freie Universität Berlin, Oertzenweg 19b, 14163 Berlin, Germany; 2 Agrarwirtschaft, FH Kiel University of Applied Sciences, Grüner Kamp 11, 24783 Osterrönfeld, Germany

**Keywords:** calcium, feed efficiency, rumen, feed additive, hypocalcemia

## Abstract

Results of recent *in vitro* experiments suggest that essential oils (EO) may not only influence ruminal fermentation but also modulate the absorption of cations like Na^+^, Ca^2+^ and NH_4_^+^ across ruminal epithelia of cattle and sheep through direct interaction with epithelial transport proteins, such as those of the transient receptor potential family. The aim of the current study was to examine this hypothesis by testing the effect of a blend of essential oils (BEO) on cation status and feed efficiency in lactating dairy cows. In the experiment, 72 dairy cows in mid-to-end lactation were divided into two groups of 36 animals each and fed the same mixed ration with or without addition of BEO in a 2×2 cross-over design. Feed intake, milk yield and composition, plasma and urine samples were monitored. Feeding BEO elevated milk yield, milk fat and protein yield as well as feed efficiency, whereas urea levels in plasma and milk decreased. In addition, plasma calcium levels increased significantly upon BEO supplementation, supporting the hypothesis that enhanced cation absorption might contribute to the beneficial effects of these EO.

## Implications

Dairy cows were offered a blend of essential oils (BEO) and its effect on cation status and performance was investigated. The study provides evidence that recently described effects of essential oils (EO) on calcium transport across the ruminal epithelium *in vitro* might be applicable *in vivo*. Supplementing certain EO may improve calcium metabolism, which could impact future hypocalcemia prevention strategies in dairy farming.

## Introduction

In recent decades, the use of EO in ruminant nutrition has received increasing attention. So far, the main focus has been on modulating ruminal microbiota. The potential of EO as antimicrobial agents was realized as early as 1965 when Borchers (1965) demonstrated that thymol arrested deamination of amino acids *in vitro* in rumen samples. Follow-up studies have since established that, at least *in vitro* and at appropriately high concentrations, EO modify fermentation patterns with effects on volatile fatty acid production, protein metabolism or both (Calsamiglia *et al.*, 2007).

At least, as interesting as the antimicrobial potential of EO is their powerful ability to modulate cellular signaling pathways in sensory perception, inflammation and pain through receptors from the transient receptor potential (TRP) family of ion channels (Holzer, 2011; Nilius and Szallasi, 2014). Interestingly, many such EO receptors (e.g. TRPA1 and TRPV3) are found in the epithelia of the intestine (Holzer, 2011), including the rumen of cattle and sheep (Rosendahl *et al.*, 2016). Recently, the impact of EO or so-called phytonutrients on ruminant immune status, insulin regulation or oxidative stress was reviewed with special notice to the involvement of TRP channels (Oh *et al.*, 2017).

Essential oils may also influence the expression patterns of other transport proteins expressed by the rumen (Mirzaei-Alamouti *et al.*, 2016). The effects of EO may thus well exceed their interaction with microbial populations in the rumen.

In cattle, the absorption of Ca^2+^ is of particular interest as low plasma Ca^2+^ levels are the cause of the metabolic disorder *milk fever* (Martín-Tereso and Martens, 2014). In many epithelia, the transport of divalent cations is known to be mediated by certain members of the TRP channel family (Wilkens *et al.*, 2009; Martens *et al.*, 2018). Intriguingly, EO have been shown to enhance the transport of Ca^2+^ and other cations across numerous preparations (Nilius and Szallasi, 2014), with studies of ruminal epithelia *in vitro* suggesting stimulatory effects on the absorption of Na^+^, NH_4_^+^ and Ca^2+^, possibly related to the expression of TRPV3 by ruminal tissue (Rosendahl *et al.*, 2016). In support of this hypothesis, it was recently demonstrated that the bovine TRPV3 mediates the transport of all three ions and that transport is stimulated by the same EO that stimulate transport across the intact ruminal epithelium (Schrapers *et al.*, 2018). This leads to the hypothesis that offering EO to cows might enhance cation absorption through TRP channels, thus facilitating the absorption of minerals and nutrients with positive effects such as improved production and health status. Accordingly, the present study focuses on the effect of a BEO on feed efficiency, nitrogen metabolism and calcium status in dairy cows.

## Material and methods

### Experimental procedures, animals and diet

The study was performed using a patented, commercial BEO (BTX12; PerformaNat GmbH, Berlin, Germany, patent US 9693971). A premix of 25 g was added to the concentrate feed, which was added to the total mixed ration (TMR). In total, animals were fed a daily dose corresponding to 1.2 g EO, with menthol as the major active compound (>80%) in addition to smaller amounts of eugenol and anethol.

The feeding trial followed a 2×2 cross-over design and was carried out at the agricultural teaching and testing institute of the state of Schleswig Holstein at Futterkamp approved by the responsible authority, the Ministry for Energy Transition, Agriculture, Environment and Rural Areas, State of Schleswig Holstein (V242-7224.121-25). A herd of 72 primi- and multiparous Holstein–Friesian cows in mid-lactation were randomly assigned to two groups which were fed either BEO or the control diet. The first BEO feeding period lasted 20 days followed by a period of 40 days in which cows of both groups were fed a standard diet without BEO to prevent carry-over effects. The second BEO feeding period of 20 days followed in which the cows that had previously been fed the control diet were fed BEO and *vice versa*.

Each group consisted of nine or 11 heifers and 25 or 27 multiparous cows, with the total mean for both groups at 2.3±0.1 lactations, 170±8 days in milk and milk yield at 37±1 kg/day at the start of the trial. Cows were milked twice daily in a milking parlour at 0600 and 1500 h. A TMR was presented directly after milking. Water and feed were available *ad libitum*. TMR consisted of grass–maize silage and concentrate feed. Samples of TMR were taken for analysis of dry matter and chemical composition. Ingredients and chemical analyses are summarized in Table 1.

**Table 1:** Diet ingredients and chemical composition of the total mixed ration for dairy cows

Feed items (% of dry matter)	
Corn silage	35.2
Grass silage	24.1
Wheat straw	2.1
Rapeseed meal	13.3
Soybean meal	3.4
Grain of maize	5.9
Dried sugar beet pulp	7.0
Rye grains	8.0
Urea	0.1
Vitamin premix	0.2
Feed lime	0.6
Cattle salt	0.2
Chemical composition (g/kg dry matter)^1^	
Dry matter	411
CP	153
Crude fat	33
Starch	179
NDF	389
ADF	217
ADL	25
Calcium	6.1
Magnesium	2.1
Phosphorous	3.8

1 Chemical composition was analysed in three samples of the total mixed ration.

### Sample collection and analysis

All data were collected both before the start and at the end of the feeding period. Individual feed intake was measured by feed weighing troughs (Insentec, Marknesse, the Netherlands) as previously described (Pahl *et al.*, 2015). In brief, troughs opened for cows identified by a transponder and closed after the individual animals left the trough. Feed intake per cow was summed up for each day. Individual milk yield was recorded at morning and afternoon milking using automatic milk counters and was summed up for daily milk yield. The last 3 days before the start and at the end of each experimental period were used to calculate individual milk yield and feed intake.

Milk fat, protein and urea were analysed twice weekly and the corresponding values for fat corrected and energy corrected milk yield (FCM and ECM) were calculated. Feed efficiency was calculated by dividing milk yield by the dry matter intake (DMI). To investigate the effect of BEO on metabolites and minerals, blood samples were collected both on the day before and on the last day of each feeding period directly after morning milking. Blood samples were taken from the coccygeal vessel using vacutainers (Greiner Bio-One International GmbH, Kremsmünster, Austria). After centrifugation, plasma was separated and stored at −20°C until analysis of metabolites and minerals. In serum samples, calcium, magnesium, phosphorus, total protein, creatinine, urea, *β*-hydroxybutyrate (BHB) and non-esterified fatty acids (NEFA) were analysed using an automatic biochemistry analyser (COBAS C-311; Roche, Mannheim, Germany). Unfortunately, due to technical problems, NEFA values were unreliable and had to be discarded.

Spontaneous urine was collected on 2 consecutive days directly after morning milking before the start and at the end of feeding period. The results of both samples were averaged to minimize daily fluctuations. In urine samples, calcium, magnesium, urea and creatinine were analysed. Urinary pH was measured immediately after collection (pH-Meter 1140; Mettler Toledo, Gießen, Germany), after which urine samples were stored at −20°C for further analysis. Urinary net acid excretion was determined in duplicate after a modified method described in Chan (1972). In brief, 10 ml urine was constantly stirred and titrated to pH of 3.5 using 1M HCl to measure titratable base. Then, samples were boiled for 2 min to remove CO_2_. After cooling down, samples were titrated to pH of 7.4 using 1 M NaOH for determination of titratable acid. To determine total ammonia concentration, acidified urine (1 ml of 4M HCl/40 ml of urine) was frozen and later analysed using an ammonia electrode via the addition method (Orion; Thermo Scientific, Bremen, Germany). Net acid excretion was calculated from titratable base minus the sum of titratable acid and ammonia. Base:acid ratio was determined by dividing titratable base by the sum of titratable acid and ammonia.

### Statistics

The effects of BEO were analysed using the univariate ANOVA procedure of SPSS 22 (SPSS Inc., Chicago, IL, USA). The samples collected before the treatment period were used as covariate. Treatment was chosen as the fixed effect and the animal was set as the random effect. Results are presented as means±SEM. Statistical differences were considered to be significant when *P*<0.05 and trends are discussed when *P*<0.10.

## Results

### Feed intake and milk parameters

Supplemental BEO did not affect feed intake (Table 2). Milk yield (milk, FCM and ECM) was significantly increased by BEO feeding. Feed efficiency (ECM/DMI and FCM/DMI) significantly increased for cows receiving BEO supplementation, milk yield per DMI tended to increase (*P*=0.066, Table 2). Fat and protein content of the milk was not influenced by BEO. Fat and protein yield were significantly higher for the BEO group (*P*=0.007 and *P*=0.003, respectively). Urea in milk significantly decreased in the BEO group (*P*=0.006, Table 2).

**Table 2:** Response of feed intake, milk parameters and feed efficiency of dairy cows following dietary supplementation with a blend of essential oils (BEO) for 20 days

Items	Control	BEO	SEM	*P*-value
DMI (kg/day)	19.7	19.5	0.28	0.558
Milk				
Milk (kg/day)	31.8	33.0	0.32	0.014
ECM (kg/day)	33.0	34.6	0.39	0.004
FCM (kg/day)	32.5	34.0	0.42	0.007
Fat (%)	3.25	3.79	0.05	0.352
Fat (kg/day)	1.15	1.23	0.02	0.007
Protein (%)	3.39	3.40	0.02	0.586
Protein (kg/day)	1.06	1.11	0.01	0.003
Urea (mg/l)	182	166	3.88	0.006
Feed efficiency				
kg milk/kg DMI	1.62	1.70	0.032	0.066
kg ECM/kg DMI	1.68	1.79	0.032	0.023
kg FCM/kg DMI	1.65	1.76	0.033	0.023

DMI=dry matter intake; ECM=energy corrected milk; FCM=fat corrected milk.

### Blood parameters

Feeding BEO was associated with a significant rise in plasma calcium levels (control: 2.46±0.015 mmol/l, BEO: 2.53±0.02 mmol/l, *P*<0.001) (Table 3). No significant changes were observed in serum levels of magnesium, phosphorus, total protein or creatinine. *β*-Hydroxybutyrate significantly decreased in the BEO group (control: 0.78±0.02 mmol/l, BEO: 0.65±0.02 mmol/l, *P*=0.001), even though cows in mid-to-late lactation state were not prone to ketosis. Plasma urea levels dropped significantly when BEO was fed (*P*<0.001, Table 3).

**Table 3:** Response of the plasma parameters of dairy cows following dietary supplementation with a blend of essential oils (BEO) for 20 days

Items (mmol/l)	Control	BEO	SEM	*P*-value
Calcium	2.46	2.53	0.02	<0.001
Magnesium	0.98	0.99	0.01	0.241
Phosphorus	1.90	1.91	0.03	0.994
Urea	4.28	3.92	0.07	<0.001
Total protein	76.0	75.5	0.32	0.215
BHB	0.78	0.65	0.02	0.001
Creatinine (µmol/l)	65.6	68.6	0.80	0.986

BHB=*β*-hydroxybutyrate.

### Urine parameters

In urine, concentrations of calcium and urea were not affected by BEO feeding (Table 4). Urinary excretion of magnesium and creatinine tended to decrease in the BEO feeding group (*P*=0.09 and 0.08, respectively). Urinary pH significantly increased (control: 8.03±0.014 mmol/l, BEO: 8.07±0.014 mmol/l, *P*=0.033). Changes in titratable acid and base were not significant, whereas net acid excretion and acid:base ratio tended to increase (*P*=0.082 and *P*=0.102, respectively). Ammonia excretion was significantly lower in the BEO group (*P*=0.013).

**Table 4:** Response of urine parameters of dairy cows following dietary supplementation with a blend of essential oils (BEO) for 20 days

Items (mmol/l)	Control	BEO	SEM	*P*-value
Calcium	1.50	1.98	0.24	0.163
Magnesium	13.6	12.6	0.40	0.090
Urea	214	211	5.99	0.772
Creatinine	7.78	7.29	0.20	0.080
pH	8.03	8.07	0.01	0.033
Titratable base	185	191	4.12	0.316
Titratable acid	70.7	68.7	1.80	0.451
NH_3_	15.6	12.5	0.88	0.013
Base:acid	2.77	2.94	0.07	0.102
Net acid excretion	99.1	109.6	4.19	0.082

## Discussion

In the current study, cows were fed 1.2 g of a commercial BEO containing menthol as the major active compound (>80%) and smaller amounts of eugenol and anethol. In response to this feed additive, an increase of milk yield, milk fat and protein yield at constant DMI was observed, resulting in a significantly elevated feed efficiency. Plasma calcium concentration increased significantly, whereas urea in plasma and milk decreased. Although this particular BEO has not been studied before, a number of other studies have addressed the effects of isolated EO or mix of these agents in feeding trials, with results highly variable and depending on the preparation studied and the study design (Khiaosa-ard and Zebeli, 2013).

In a recent study of cows fed a ketogenic diet, a commercial BEO significantly increased milk fat content, energy-corrected milk and feed efficiency (ECM/DMI) (Drong *et al.*, 2016). However, this occurred with a concomitant rise in BHB and NEFA, both of which rose more strongly when EO were added to the ketogenic diet, suggesting a negative impact of the preparation on energy balance in these challenged animals. In a study of cows fed a more balanced diet, BHB and NEFA were not affected by this blend (Tassoul and Shaver, 2009), which is in contrast to the results of this study showing decreased BHB values in cows offered BEO feeding. However, more work is clearly needed to assess the impact of EO on fat mobilization and energy balance of lactating cows, in particular in the transition period.

An interesting observation in this study was the significant drop in plasma urea levels, which most likely explains the concomitant lower milk urea levels during BEO supplementation. As milk urea nitrogen (MUN) can be used to predict total urinary nitrogen excretion (Nousiainen *et al.*, 2004; Spek *et al.*, 2013), a reduction in total nitrogen excretion can be inferred. A drop in MUN was also observed in a study of ewes fed another commercial BEO (Giannenas *et al.*, 2011), whereas in lactating cows, MUN values that were elevated by monensin supplementation were normalized (Benchaar *et al.*, 2006). As daily milk protein yield increased, nitrogen might have been incorporated more efficiently into protein and amino acids such as glutamine rather than non-protein nitrogen like urea.

To the extent that they have been validated, the mechanisms behind the *in vivo* effects of EO are thought to reflect the well-documented antimicrobial effects of EO (Borchers, 1965; Calsamiglia *et al.*, 2007). Various mechanisms for the bactericidal effects of these compounds are discussed, such as an interaction with the lipid membrane itself or proteins embedded therein, ultimately leading to a breakdown in membrane barrier (Jouany and Morgavi, 2007) with a differential impact on Gram-negative and Gram-positive bacteria (Chao *et al.*, 2000).

However, in particular, at the concentrations fed *in vivo*, the mode of action of EO fed to dairy cows may exceed the antimicrobial effects. We found higher plasma calcium levels – a fact that might relate to the well-documented stimulatory interaction of EO with non-selective cation channels from the TRP channel family (Holzer, 2011; Nilius and Szallasi, 2014; Rosendahl *et al.*, 2016; Schrapers *et al.* 2018). Effects of EO on the transport of various ions across epithelia of the gastrointestinal tract have been reported not only in human and rat colon (Kaji *et al.*, 2011) but also notably in the ruminal epithelium of cattle and sheep (Rosendahl *et al.*, 2016). Furthermore, we were able to show that menthol and thymol stimulated the transport not just of Na^+^, but also of NH_4_^+^ and Ca^2+^ across the isolated ruminal epithelia of sheep and cattle in an *ex vivo* approach (Rosendahl *et al.*, 2016). In addition to these acute effects, a change in the expression pattern of acid base transporting proteins was recently shown in the ruminal epithelium of sheep fed a blend of extracts from a variety of plants that included peppermint (Mirzaei-Alamouti *et al.*, 2016).

Both menthol and thymol stimulate certain non-selective cation channels of the TRP family that are expressed by the tissue (Nilius and Szallasi, 2014; Rosendahl *et al.*, 2016). With due caution, the low-dose BEO fed in this study may have stimulated the transport of calcium from rumen into blood, which might explain the increase of plasma Ca^2+^ observed. An increased ruminal uptake of calcium should enhance calcium homeostasis, with particular relevance for the reduction of hypocalcemia around calving (Goff, 2008).

Based on our *in vitro* study (Rosendahl *et al.*, 2016), we initially expected to see higher absorption of ammonia as NH_4_^+^ from the rumen, and thus higher plasma and milk urea levels in the feeding group receiving EO. However, two factors may have intervened. First, degradation of protein may have been reduced by inhibition of proteolytic bacteria (McIntosh *et al.*, 2003). Second, given the very high pK value of the strong base ammonia (NH_3_) of 9.2, a shift away from the absorption of NH_3_ and towards the absorption of the protonated cationic form (NH_4_^+^) through a TRP channel can be expected to induce a shift in the acid–base equilibrium of the blood towards less alkaline values. A decrease in pH will cause an activation of glutamine synthetase in the liver (Taylor and Curthoys, 2004; Weiner and Verlander, 2017). This normalizes the blood pH as the protons, which would be released in the course of urea synthesis are incorporated into the glutamine molecule. In the form of the non-toxic glutamine, protons and ammonia can then be transferred to the kidney and excreted, which may explain the higher net acid excretion observed under the BEO in this study.

As the most abundant amino acid in plasma, glutamine plays a central role not only as a shuttle for ammonia and protons but also as a key player in protein metabolism (Taylor and Curthoys, 2004), with the supply limited in stress situations such as calving (Meijer *et al.*, 1993). An increase in glutamine synthesis from ammonium leaving the rumen would certainly appear to facilitate protein metabolism in animals challenged by lactation, which might be supported by the reduced NH_3_ excretion in cows fed BEO. The possibility that certain BEO have the capacity not only to enhance proton absorption from the rumen but also to shift ammonia detoxification towards glutamine production is speculative. This hypothesis clearly merits further investigation.

Currently, the assumption that beneficial effects of feeding EO are caused by influencing the gastrointestinal flora represents the most established hypothesis. However, the ability of certain EO to affect epithelial ion transport *in vitro* might also contribute to their *in vivo* effects. This approach might provide an explanation for the high variability in the outcome of studies, as the effects of EO on epithelial ion transport are highly dependent on the dose and the substance. Further research is needed to elucidate the complex interaction of EO, gastrointestinal bacteria and the ruminal epithelium, contributing to the multiple observed positive effects on milk yield, feed efficiency and possible impacts on calcium homeostasis.

In conclusion, the tested BEO increased feed efficiency and plasma calcium levels. Possibly, this reflects stimulatory effects of EO on ruminal calcium absorption, which might help to cover the increased calcium demand during lactation.

## Conclusion

The blend of plant bioactive EO tested in this study had effects that suggest improved feed efficiency and calcium homeostasis. We propose that in addition to their effects on fermentational patterns; EO activate specific cation-transporting proteins expressed by the rumen, resulting in an increased uptake of cations like calcium and ammonium. Further studies are required to elucidate the fate of the absorbed nutrients with special regard to calcium and nitrogen.

## References

[ref2] Benchaar C, Petit HV, Berthiaume R, Whyte TD and Chouinard PY 2006 Effects of addition of essential oils and monensin premix on digestion, ruminal fermentation, milk production, and milk composition in dairy cows. Journal of Dairy Science 89, 4352–4364. 17033023 10.3168/jds.S0022-0302(06)72482-1

[ref3] Borchers R 1965 Proteolytic activity of rumen fluid. Journal of Animal Science 24, 1033–1038.

[ref4] Calsamiglia S, Busquet M, Cardozo PW, Castillejos L and Ferret A 2007 Invited review: essential oils as modifiers of rumen microbial fermentation. Journal of Dairy Science 90, 2580–2595. 17517698 10.3168/jds.2006-644

[ref6] Chan JCM 1972 The rapid determination of urinary titratable acid and ammonium and evaluation of freezing as a method of preservation. Clinical Biochemistry 5, 94–98. 5039597 10.1016/s0009-9120(72)80014-6

[ref7] Chao SC, Young DG and Oberg CJ 2000 Screening for inhibitory activity of essential oils on selected bacteria, fungi and viruses. Journal of Essential Oil Research 12, 639–649.

[ref8] Drong C, Meyer U, von Soosten D, Frahm J, Rehage J, Breves G and Dänicke S 2016 Effect of monensin and essential oils on performance and energy metabolism of transition dairy cows. Journal of Animal Physiology and Animal Nutrition 100, 537–551. 26613964 10.1111/jpn.12401

[ref9] Giannenas I, Skoufos J, Giannakopoulos C, Wiemann M, Gortzi O, Lalas S and Kyriazakis I 2011 Effects of essential oils on milk production, milk composition, and rumen microbiota in Chios dairy ewes. Journal of Dairy Science 94, 5569–5577. 22032380 10.3168/jds.2010-4096

[ref10] Goff JP 2008 The monitoring, prevention, and treatment of milk fever and subclinical hypocalcemia in dairy cows. The Veterinary Journal 176, 50–57. 18342555 10.1016/j.tvjl.2007.12.020

[ref11] Holzer P 2011 TRP channels in the digestive system. Current Pharmaceutical Biotechnology 12, 24–34. 20932260 10.2174/138920111793937862 PMC3160477

[ref12] Jouany J-P and Morgavi DP 2007 Use of ‘natural’ products as alternatives to antibiotic feed additives in ruminant production. Animal 1, 1443–1466. 22444918 10.1017/S1751731107000742

[ref13] Kaji I, Karaki S and Kuwahara A 2011 Effects of luminal thymol on epithelial transport in human and rat colon. American Journal of Physiology-Gastrointestinal and Liver Physiology 300, G1132–G1143. 21372164 10.1152/ajpgi.00503.2010

[ref14] Khiaosa-ard R and Zebeli Q 2013 Meta-analysis of the effects of essential oils and their bioactive compounds on rumen fermentation characteristics and feed efficiency in ruminants. Journal of Animal Science 91, 1819–1830. 23345564 10.2527/jas.2012-5691

[ref16] Martens H, Leonhard-Marek S, Röntgen M and Stumpff F 2018 Magnesium homeostasis in cattle: absorption and excretion. Nutrition Research Reviews (1–17, doi:10.1017/S0954422417000257, Published online by Cambridge University Press 10 January 2018. 10.1017/S0954422417000257 29318981

[ref17] Martín-Tereso J and Martens H 2014 Calcium and magnesium physiology and nutrition in relation to the prevention of milk fever and tetany (dietary management of macrominerals in preventing disease). Veterinary Clinics of North America: Food Animal Practice 30, 643–670. 25245611 10.1016/j.cvfa.2014.07.007

[ref18] McIntosh FM, Williams P, Losa R, Wallace RJ, Beever DA and Newbold CJ 2003 Effects of essential oils on ruminal microorganisms and their protein metabolism. Applied and Environmental Microbiology 69, 5011–5014. 12902303 10.1128/AEM.69.8.5011-5014.2003 PMC169102

[ref19] Meijer GA, van der Meulen J and van Vuuren AM 1993 Glutamine is a potentially limiting amino acid for milk production in dairy cows: a hypothesis. Metabolism 42, 358–364. 8487655 10.1016/0026-0495(93)90087-5

[ref20] Mirzaei-Alamouti H, Moradi S, Shahalizadeh Z, Razavian M, Amanlou H, Harkinezhad T, Jafari-Anarkooli I, Deiner C and Aschenbach JR 2016 Both monensin and plant extract alter ruminal fermentation in sheep but only monensin affects the expression of genes involved in acid-base transport of the ruminal epithelium. Animal Feed Science and Technology 219, 132–143.

[ref21] Nilius B and Szallasi A 2014 Transient receptor potential channels as drug targets: from the science of basic research to the art of medicine. Pharmacological Reviews 66, 676–814. 24951385 10.1124/pr.113.008268

[ref22] Nousiainen J, Shingfield KJ and Huhtanen P 2004 Evaluation of milk urea nitrogen as a diagnostic of protein feeding. Journal of Dairy Science 87, 386–398. 14762082 10.3168/jds.S0022-0302(04)73178-1

[ref23] Oh J, Wall EH, Bravo DM and Hristov AN 2017 Host-mediated effects of phytonutrients in ruminants: a review. Journal of Dairy Science 100, 5974–5983. 28390713 10.3168/jds.2016-12341

[ref24] Pahl C, Hartung E, Mahlkow-Nerge K and Haeussermann A 2015 Feeding characteristics and rumination time of dairy cows around estrus. Journal of Dairy Science 98, 148–154. 25465539 10.3168/jds.2014-8025

[ref26] Rosendahl J, Braun HS, Schrapers KT, Martens H and Stumpff F 2016 Evidence for the functional involvement of members of the TRP channel family in the uptake of Na^+^ and NH_4_ ^+^ by the ruminal epithelium. Pflügers Archiv-European Journal of Physiology 468, 1333–1352. 27184746 10.1007/s00424-016-1835-4

[ref27] Schrapers K, Sponder G, Liebe F, Liebe H and Stumpff F 2018 The bovine TRPV3 as a pathway for the uptake of Na^+^, Ca^2+^, and NH_4_ ^+^ . PlosOne 13, e0193519. 10.1371/journal.pone.0193519 PMC5832270 29494673

[ref28] Spek JW, Dijkstra J, Van Duinkerken G, Hendriks WH and Bannink A 2013 Prediction of urinary nitrogen and urinary urea nitrogen excretion by lactating dairy cattle in northwestern Europe and North America: a meta-analysis. Journal of Dairy Science 96, 4310–4322. 23664347 10.3168/jds.2012-6265

[ref29] Tassoul MD and Shaver RD 2009 Effect of a mixture of supplemental dietary plant essential oils on performance of periparturient and early lactation dairy cows. Journal of Dairy Science 92, 1734–1740. 19307655 10.3168/jds.2008-1760

[ref30] Taylor L and Curthoys NP 2004 Glutamine metabolism: role in acid-base balance. Biochemistry and Molecular Biology Education 32, 291–304. 21706743 10.1002/bmb.2004.494032050388

[ref33] Weiner ID and Verlander JW 2017 Ammonia transporters and their role in acid-base balance. Physiological Reviews 97, 465–494. 28151423 10.1152/physrev.00011.2016 PMC5539407

[ref34] Wilkens MR, Kunert-Keil C, Brinkmeier H and Schröder B 2009 Expression of calcium channel TRPV6 in ovine epithelial tissue. The Veterinary Journal 182, 294–300. 18701326 10.1016/j.tvjl.2008.06.020

